# Objective risk and protective factors for momentary and daily loneliness:using digital phenotyping and temporal analysis

**DOI:** 10.1038/s44184-025-00148-4

**Published:** 2025-09-07

**Authors:** Matthias Haucke, Stephan Heinzel, Shuyan Liu

**Affiliations:** 1 Department of Psychiatry and Psychotherapy, Charité – Universitätsmedizin Berlin (Campus Charité Mitte), Berlin, Germany; 2 Departement of Educational Sciences and Psychology, Clinical and Biological Psychology, Technische Universität Dortmund, Dortmund, Germany; 3 German Center for Mental Health (DZPG), Berlin, Germany

**Keywords:** Psychology, Medical research

## Abstract

Loneliness is a growing global health issue, yet real-time assessments of its objective risk and protective factors are limited. This study identifies momentary and daily predictors using digital phenotyping and temporal analysis. Analyzing 12788 momentary observations from social mobile sensing and actigraphy, we examined how they impact loneliness on average (between-person) and in daily fluctuations (within-person). Instant messenger app usage was associated with increased loneliness, both momentarily (B = 2.95, *p* = 0.017) and daily (B = 2.83, *p* = 0.018), within and between subjects. Social media usage was associated with higher within-person momentary loneliness (B = 0.53, *p* = 0.001). An exploratory network analysis suggested that physical activity is associated with in-person social interaction, which is negatively associated with loneliness, while social media may replace social interactions. Thus, objective risk factors include increased use of social media and instant messaging, whereas greater physical activity may serve as a protective factor.

## Introduction

Loneliness has become a pressing public health concern, affecting 10% of European adults and is linked to adverse physical and mental health outcomes^1–3^. Despite its high prevalence and serious consequences, loneliness remains poorly understood, particularly in terms of its individual risk factors^4,5^. Digital phenotyping offers a promising approach for identifying such factors by capturing objective, real-time behavioral and physiological data in naturalistic settings^6^. This method assesses and estimates human behavior and emotional states through data captured from smartphones and wearable devices (e.g. physical activity levels, phone call duration, and social app usage)^5^. In this study, we examine how risk and protective factors, derived from smartphones and wearable actigraphy devices, affect momentary and daily loneliness. In addition, we apply a network approach^7^ to investigate the temporal dynamics between momentary loneliness, social activity, and digital phenotyping.

A core motivation underlying human thought, emotion, and behavior is the intrinsic need to form meaningful relationships^8^. When there is a gap between one’s actual and desired interpersonal relationships, loneliness emerges^9^. As a subjective experience loneliness differs from an objective count of social interaction. In other words, one can feel lonely even in the presence of others, and conversely, feel connected while being alone^10^. Loneliness has a profound impact on mental health and is strongly associated with a range of psychiatric disorders, including depression^1,2^, schizophrenia^11,12^ and anxiety disorders^13,14^. Moreover, the impact of loneliness on mortality is comparable to smoking 15 cigarettes a day and surpasses the risk associated with obesity^15^.

Traditionally, loneliness has been assessed using self-reports, such as the University of California Los Angeles (UCLA) Loneliness Scale, which can be time-consuming and subject to reporting and recall biases^16,17^. In addition, the subjective measure of loneliness has several shortcomings. First of all, loneliness is subject to social stigma, which can prevent individuals from acknowledging or describing themselves as lonely^18^. Moreover, people can vary in their ability to recognize and assess their emotional states, including loneliness^19^. Advancements in digital technology enable to identify objective within-person risk factors that may predict subjective experiences of loneliness. This non-invasive approach enables passive data collection without requiring active engagement^20^, which is crucial for real-time detection. This enables the detection of loneliness before it reaches harmful levels, thereby facilitating timely and targeted interventions^21^.

Objective within-person risk factors for loneliness could be derived from wearable devices, such as actigraphy monitors worn on the wrist, hip, or ankle, which provide data on sleep patterns and physical activity (PA)^22^. PA refers to any form of body movement including exercise and sports^23^. A meta-analysis^24^ has shown a negative association between PA and feelings of loneliness, although the underlying mechanisms and temporal directionality remains unclear. Does loneliness reduce physical activity, or does physical activity reduce loneliness? PA may help alleviate loneliness by facilitating social interactions, such as those during group exercise classes^24,25^. Furthermore, engaging in outdoor activities in nature, rather than remaining sedentary at home, may alter the context in which loneliness is experienced, potentially reducing it^26^.

Another potential objective indicator of loneliness that can be derived from actigraphy devices is sleep efficiency, defined as the ratio of time spent asleep to time spent lying in bed^27^. Sleep efficiency is a vital component of well-being, closely linked to both physical and mental health, as it affects physiological processes, including nervous system activity, hormonal regulation and inflammation^28^. In controlled laboratory settings, individuals who are lonely, compared to those who are not, exhibited lower sleep efficiency and spent more time awake after sleep onset, indicating poorer overall sleep quality^29^. In line with this, a meta-analysis^30^ has found a medium-sized correlation between loneliness and sleep disturbances, although the directionality of this relationship remains unclear. Some theories propose that poor sleep quality contributes to increased social withdrawal, potentially creating a vicious cycle of loneliness and sleep deprivation^31^. Other theories suggest that this relationship may stem from heightened stress and hypervigilance associated with social isolation, disrupting the body’s ability to relax and enter restorative stages of sleep^29^. Yet, empirical evidence supporting this reciprocal relationship is lacking. Monitoring sleep through wearable devices provides valuable insights into the temporal dynamics of how loneliness influences objectively measured sleep efficiency in real-world settings.

An alternative approach to objectively estimating loneliness is social mobile sensing, which relies on metadata from smartphones, such as app usage, call duration, and SMS messages^16^. This data reflects social activity and has been analyzed with machine learning to identify individuals experiencing loneliness^22,32–34^. For example, Jafarlou et al.^35^ used smartphones to track behavioral patterns, such as location changes, notifications, phone calls, and text messages, as well as smartwatches and smart rings, to monitor physiological and sleep-related metrics. They demonstrated that machine learning models can detect loneliness with an accuracy of up to 82%. While machine learning can predict loneliness episodes, it is less effective at explaining the complex interactions and temporal directionality of factors derived from digital phenotyping^36^. However, understanding these dynamics is crucial for designing effective interventions. For example, understanding whether social media use increases or decreases subsequent social interactions is essential for determining whether it can serve as a reliable trigger for a loneliness intervention. Previous studies indicate that social mobile sensing may be negatively associated with loneliness. Increased telephone usage^37^, Facebook activity and WhatsApp usage have been associated with reduced feelings of loneliness^38^. However, these associations may be influenced by factors such as participant’s gender and age^16^. In addition most existing studies rely on one-time, survey-based self-reports of loneliness (for a comprehensive review, see Qirtas, et al.^5^), which provide limited insight into the association between digital phenotyping and within-person, moment-to-moment fluctuations of loneliness.

To investigate the real-time impact of objective markers of loneliness derived from social mobile sensing and actigraphy devices, we employ a temporal dynamic network approach^39^. This approach posits that changes in mental states arise from dynamic interactions between mental states and behaviors over time^7^. By using this method, we can examine moment-to-moment time-lagged associations between digital phenotyping, social mobile sensing, and loneliness. For example, social media usage at 10 a.m. may lead to increased loneliness at 1 p.m. This network approach allows us to gain a more nuanced understanding of how digital phenotyping and loneliness influence each other over time.

In sum, our study aims to identify objective risk factors that can estimate momentary and daily loneliness, using smartphone mobile sensing and wearable devices. In addition, we explore the temporal dynamics between risk factors and loneliness. Specifically, we hypothesize that increased physical activity, improved sleep efficiency, and increased social mobile sensing (e.g. phone calls, social app usage) will be negatively associated with momentary and daily loneliness. Given limited prior research, we explored the temporal relationships between momentary loneliness, social interactions, and digital phenotyping without forming specific a priori hypotheses.

## Methods

### Participants and Procedure

**Fig. 1:**
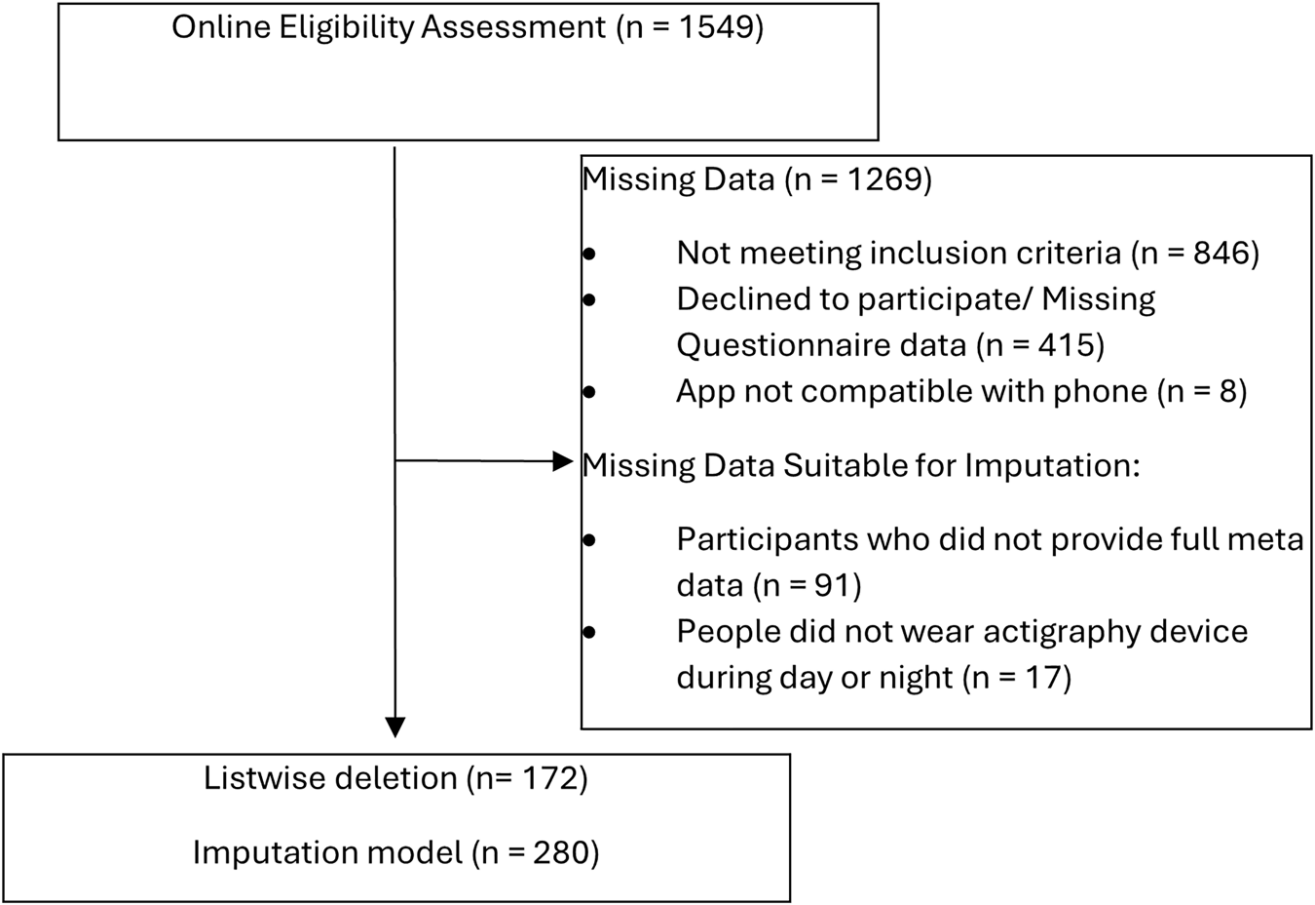
Recruitment flow. The recruitment flow that led to the day-level sample assuming listwise deletion (*n* = 172), utilizing EMA, mobile sensing and wearable devices.

**Fig. 2:**
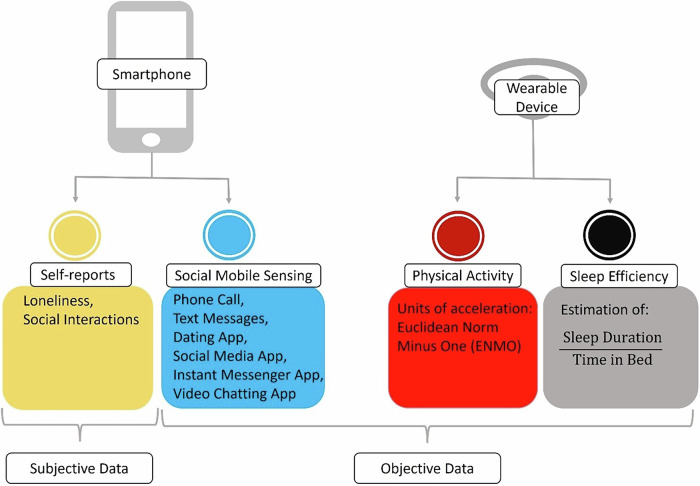
Overview of the collected data. Subjective data includes self-reports of loneliness and social interactions. Objective data includes social mobile sensing, physical activity, and sleep efficiency, measured by smartphone and wearable devices.

We screened a total of 1549 participants between August 2020 and March 2021 for this ecological momentary assessment (EMA) study. Participants were recruited across Germany through advertisements on university websites, eBay classifieds, and social media. To screen participants, we used the Siuvo Intelligent Psychological Assessment Platform. Eligibility criteria were: (1) being at least 18 years old, (2) not working in night shifts, (3) using an Android smartphone (due to App compatibility), (4) providing informed consent and (5) speaking fluent German. Additionally, participants needed to report at least occasional feelings of loneliness (measured by the 8-item UCLA Loneliness Scale (ULS-8); cutoff score = 16^40^) and mild distress (assessed by the COVID-19 Peritraumatic Distress Index (CPDI); cutoff score = 28^41^). Participants wore an actigraphic device, called “GENEActiv” (Activinsights) (dynamic range ±8 g, sampling frequency range 10–100 Hz), on their left wrist. As shown in Fig. 1, 189 out of 280 participants (67.5%) agreed to transfer the required smartphone metadata. Of these 189, 172 participants (91%) wore their actigraphy device during day and night. To compensate participants, we provided a financial reward of €70. The study received approval from the Ethics Committee of Charité – Universitätsmedizin Berlin (ref: EA2/143/20) and Freie Universität Berlin (ref: 030/2020). In this study, we passively collected smartphone metadata and sent participants questionnaires about their social activity and loneliness. Additionally, we employed wearable actigraphy devices to collect data on physical and sleep activity. Therefore, we gathered subjective data via self-reported questionnaires as well as objective data by passively collecting metadata on social activity, as well as actigraphy data on sleep efficiency and physical activity (see Fig. 2).

### Ecological momentary assessment

We used a smartphone application “movisensXS” (movisens GmbH, Karlsruhe, Germany). This app allowed us to passively gather smartphone meta data and send questionnaires about social activity and loneliness to participants. Participants completed questionnaires for 7 consecutive days, receiving a total of 8 randomized prompts each day. These prompts were randomly distributed within blocks of time lasting 1 hour and 45 min, with a minimum break of 30 min between each assessment, between 8 AM and 10 PM. The beep was delivered either as an alarm tone or a vibration, depending on the settings of the participant’s mobile phone. Participants were encouraged to use the vibration mode, as it was less likely to interfere with their daily activities.

Duration of social activity was measured with the question “During the last hour, how long did your last social contact last?” via a Likert scale ranging from 1 = “0 min” to 7 = “50–60 min”. Momentary loneliness was measured with the question “During the last hour, to which extent did you feel lonely?”, on a VAS (0–100: 0 = not at all, 100 = extremely). Daily loneliness was measured once in the evening with the 3-item UCLA Loneliness scale (ULS-3)^42^, on a VAS (0–100: 0 = not at all, 100 = extremely).

During the study, the application gathered data on the usage time of social communication applications, short message service (SMS) and phone calls. Usage time refers to the time people were actively using a social communication application in minutes. Except for the category “text messages”, for which we counted the length of short message services (SMS). We categorized the smartphone communication data into six types in line with previous studies^16,43,44^: 1. Social media (Facebook, Instagram and Snapchat), 2. Instant messenger (WhatsApp, Telegram, Facebook Messenger, and Skype Messenger), 3. Video Calls (Skype and Zoom). In addition, we added a fourth app category: 4. Dating (Bumble, Tinder, Grindr). Finally, we added 5. Phone calls and 6. SMS as categories.

Physical activity and sleep efficiency were collected via the actigraphy device GENEActiv (Activinsights) (dynamic range ±8 gravitational units (g) sampling frequency range 10–100 Hz). Participants wore these devices at their non-dominant wrist.

### Statistical analysis

We conducted our statistical analyses in the R version 4.4.1 system (www.r-project.org). We conducted two levels of analysis: one at the day level and another at the momentary level. To ensure comparability among the predictors, we first aligned all social interaction and social mobile sensing on the same time scale. Specifically, we converted the timestamps into 10 min intervals by first converting seconds to minutes and then dividing the resulting values by 10. Next, we applied z-standardization to each predictor, allowing for direct comparison of effect sizes. In addition, we controlled both between-person and within-person effects^45^. To achieve this, each Level 1 predictor was person-mean centered (e.g., fluctuations in instant messaging usage around an individual’s average) to capture within-person variability. Corresponding person-level averages (e.g., a person’s average instant messaging usage) were then included as Level 2 predictors to account for between-person differences. For both analyses, we estimated a multilevel hierarchical regression model with the outcome variable “loneliness” using the Akaike Information Criterion for model selection^46^. Model selection procedure showed that the best fit for our data was a random intercept model for a day and momentary level (see Supplementary Table 1).

Due to the presence of missing data, we employed an imputation method to reduce potential bias and improve model accuracy^47^. The missing data visualization (see Supplementary Fig. 1) indicates that overall missingness is relatively low (7%) and concentrated in a few key variables, notably Sleep Efficiency (24%), ENMO scores (11%), and Phone Call Duration and SMS Length (each 31%). Phone Call Duration and SMS Length were missing when participants who did not provide extra consent to collect this type of mobile communication data. Additionally, Sleep Efficiency and ENMO values were missing, when participants did not wear their device during the night or day. This indicates that listwise deletion is not appropriate, as it assumes data is Missing Completely At Random (MCAR)^48^. In our dataset most other variables, including central predictors like Gender and Weekday are fully observed. This pattern suggests that multiple imputation is more appropriate, as it can effectively recover information by leveraging a rich set of non-missing variables^48^. A sensitivity analysis, in which we compare the imputation results with the list-wise deletion method can be found in Supplementary Table 2.

Specifically, we imputed missing data by following the procedure by Van Buuren 2018^49^. The R package MICE (Multiple Imputation via Chained Equations) was used to fill in missing data through an iterative series of predictive models (m = 100). Level-1 variables, including loneliness, duration of social contact, social mobile sensing, physical activity, and sleep efficiency, were imputed using two-level predictive mean matching. This approach leveraged cluster means of covariates from other Level-1 variables, along with Level-2 variables, to inform the imputation. Moreover, level- 2 variables (Age, Lockdown Stage and Gender) were imputed by predictive mean matching, using predictors at the aggregated level-1 (e.g. loneliness) and level-2 (e.g., Gender). Our imputed missing data approximately matched the shape of our observed data (See Supplementary Fig. 2).

After the model selection procedure, we controlled for Age, Gender (Female, Male, Diverse), whether there was a Lockdown stage (Lockdown, No-lockdown) and Weekday (Monday-Sunday). As some of the model assumptions were violated (see Supplementary Fig. 3), we used a bootstrap procedure with 1000 repetitions, to estimate the 95% confidence intervals (CIs) for each parameter. Bootstrapping helps to address violations of multilevel regression assumptions by providing robust, assumption-free estimates of standard errors and confidence intervals through empirical resampling^50^. The R code and data can be found online: https://osf.io/rs2nd/. The hypotheses and analyses were not preregistered.

### Data preparation

Participants were excluded if they completed fewer than 30% of the daily and momentary assessments during the study period. For the day-level analysis we excluded 2 participants. For the momentary analysis we excluded 1 participant.

We screened the app usage protocol for the usage of apps according to the specified 4 social communication categories: 1. Social media, 2. Instant messenger 3. Video Calls and 4. Dating app. Examples of the raw data from the app usage protocol can be found in Supplementary Table 3.

On the day level, we calculated the daily sum score for app usage time and phone call duration. In addition, we calculated the length of each received or send SMS per day. To minimize potential measurement errors, we compared the extracted app usage data with a device running log. We excluded app usage metadata whose time frames fell outside 2 standard deviations above the mean device running time. As a result 26 observations were excluded.

To estimate physical activity, we calculate the daily average (24 h) of the Euclidean Norm Minus One with negative values rounded up to zero (ENMO). Euclidean norm (vector magnitude) is a validated summary score of body acceleration of the raw signals of three-measurement axis^51^. The Euclidean norm minus one (ENMO) is defined as ri–1000 ^52^, where

1 $$\,{ri}=\sqrt{{x}_{i}^{2}+{y}_{i}^{2}+{z}_{i}^{2}}={i}^{{th}}{vector\; magnitude\; at\; each\; timepoint}$$

The actigraphy data from GENEActiv (100 Hz;.bin files) were downloaded using GENEActiv PC software V3.3. The GENEActiv.bin files were then exported into R statistical software V4.4.1 for processing using the GGIR package V1.2-0. We autocalibrated the raw triaxial accelerometer signals and computed the average ENMO metric for each day. To visually check for potential measurement errors, we displayed the calculated ENMO scores in a boxplot, we saw that there were 3 outliers that were above 2 standard deviations from the average ENMO score, which were excluded from the analyses, see Supplementary Fig. 4.

To determine sleep efficiency from the actigraphy device data, we used the default settings of the GGIR package. The GGIR package first distinguishes between sustained inactivity and wakefulness periods. Then time windows that guide sleep detection are identified. Finally, sleep period time windows (SPT) are defined based on the previous steps. The HDCZA algorithm is used to estimate SPT. This algorithm, designed for studies using wrist-worn accelerometers without a sleep log, is described in more detail by Van Hees et al.^27^. The HDCZA algorithm classifies periods with limited posture changes and identifies the longest block of the day to represent the guider window. In the GGIR package, sleep periods are referred to as sustained inactivity bouts (SIB). Sleep efficiency was calculated using the formula:

2 $$\mathrm{Sleep}\,\mathrm{Efficienc}y\text{=}\frac{\mathrm{Sleep}\,\mathrm{Duration}}{\mathrm{Time}\,\mathrm{in}\,\mathrm{Bed}}$$

where, “Sleep Duration” equals the accumulated nocturnal sustained inactivity bouts within the Sleep Period Time and “Time in Bed” is the time difference between getting into and getting out of bed. Moreover, the GGIR packages calculates the non-wear percentage during the SPT of a given day.

To decrease measurement error due to participants who did not wear their device during the night, we excluded 45 data points for which the HDCZA algorithm could not estimate SPT. Moreover, we excluded 5 data points with a non-wear percentage during the SPT of 50% or higher, indicating that the participant did not wear the actigraphy device for at least half of the time during SPT. Additionally, one data point for sleep efficiency, which had a value of 700, was excluded as a measurement error because it significantly exceeded the maximum value of 1. Finally, when examining the calculated sleep duration, we excluded 56 data points that were more than 2 standard deviations above or below the average sleep duration (7 h ± 3.8 h). Refer to Supplementary Fig. 4 for more details.

For the momentary analysis, we computed the average ENMO metric for one hour prior to each momentary loneliness assessment (beep). To exclude times in which participants did not wear their actigraphy device, we used the nonwear score of the GGIR package (cut-off score = 1). Additionally, we calculated the total duration of phone calls, the lengths of SMS messages, the time spent using dating apps, social media, instant messaging, and video chatting—all for one hour prior to each beep (see Supplementary Fig. 5).

As a result of this procedure, the number of data points varied across mobile sensing categories at the momentary level. Notably, there were no data points for dating app usage, as well as very few observations for SMS length 1 h prior to each momentary beep. Furthermore, messenger app usage and social media usage were most frequently used one hour before each beep (see Supplementary Fig. 6). Due to missing observations the dating category was not included in the momentary-level analysis.

For the momentary network analysis, we built a lag 1 multilevel vector autoregressive model (mlVAR) using the R package mlVAR. In this mlVAR model, each variable at time point *t* was predicted by all variables (including itself) at the previous time point (lag 1). The results of the network models comprised nodes (representing variables) and directed edges (indicating statistical relationships), and were visualized via the R package qgraph^53^. Edges are color-coded to represent their nature: red edges indicate negative associations, whereas blue edges signify positive ones. The thickness of each edge corresponds to the strength of the association, with thicker edges reflecting stronger relationships^54^. Only statistically significant edges, defined by a significance level of *p* < 0.05, are included in the visual representation.

To test for stationarity, we conducted a Kwiatkowski-Phillips-Schmidt-Shin (KPSS) test individually for each subject and variable. The results indicated that the data were stationary in approximately 97% of cases.

## Results

### Demographics

Our analysis consists of 280 participants, including 31% male, 1% diverse and 68% female participants. The participants had an average age of 30.85 years (SD = 11.27), ranging from 18 to 72 years. Their mean loneliness level, as assessed via the UCLA-8 scale was 22.59 (SD = 3.98) (UCLA-8 ranges from 8 = Never to 32 = Always). To examine the relationship between demographic variables and loneliness, we conducted Pearson correlation analyses (r) for continuous and ANOVA for categorical variables. For the statistical analysis, we calculated an average score for each participant. Household size was negatively correlated with loneliness (*r* = −0.14, *p* = 0.022), suggesting that larger household size is associated with lower loneliness. The ULS-8 loneliness score showed a moderate positive correlation with daily ULS-3 loneliness scores (r = 0.32, *p* < 0.001). Duration of social interaction was negatively correlated with loneliness (r = −0.25, *0* < 0.001). All participants in our sample reported experiencing loneliness at least occasionally, to ensure that both groups reflect individuals with some level of loneliness. The sample characteristics and their correlations with loneliness are summarized in Table 1.

**Table 1:** Demographics, predictors and their correlations with loneliness before imputation

Variable	Type of Data (Subjective self-reports/Objective mobile sensing)	Total (n = 280) Mean (SD)/Sum (Percentage)	Statistical analysis (Pearson correlation (r), ANOVA, t-test, chi-square test)
**Baseline**			
Age (in years), mean (SD)	Subjective	30.85 (11.27)	*r* = *0.01, p* = *0.80*
Gender, n (%)	Subjective	Female = 190 (68%), Male = 87 (31%), Diverse = 3 (1%)	*F*(2277) = 1.02, *p* = 0.363,
Family Status	Subjective	Single = 125 (45%), In partnership = 98 (35%), Married = 52 (19%), Other = 4 (1%)	*F*(1277) = 3.38, *p* =0.067
Number of children, mean (SD)	Subjective	0.43 (0.85)	*r* = −*0.09, p* = *0.15*
Household size, number of people mean (SD)	Subjective	2.5 (2.1)	***r*** = −***0.14, p*** = ***0.022***
ULS-8 Loneliness score, sum (SD) (ranging from 8 = Never to 32 = Always)	Subjective	22.59 (3.98)	***r*** = ***0.32, p*** < ***0.001***
**Smartphone-based Day Level**			
ULS-3 Loneliness score, mean (SD),(VAS; 0–100: 0 = not at all, 100 = extremely).	Subjective	34.82 (23.42)	***r*** = ***1, p*** < ***0.001***
Duration Social Interaction (7-point Likert scale; 1 = “0 minutes” to 7 = “50–60 minutes”), mean (SD)	Subjective	1.96 (1.4)	***r*** = –***0.25, p*** < ***0.001***
Phone Call Duration (in minutes), mean (SD)	Objective	13.56 (20)	*r* = *0.11, p* = *0.127*
SMS Length (number of characters), mean (SD)	Objective	89.28 (176.86)	*r* = *0.07, p* = *0.36*
Dating App Duration (in minutes), mean (SD)	Objective	1.15 (4.45)	*r* = *0.01, p* = *0.81*
Social Media Duration (in minutes), mean (SD)	Objective	26.53 (36.94)	*r* = *0.012, p* = *0.836*
Instant Messenger Duration (in minutes), mean (SD)	Objective	29.47 (34.32)	*r* = *0.11, p* = *0.07*
Video Chatting Duration (in minutes), mean (SD)	Objective	0.41 (2.38)	*r* = *0.05, p* = *0.41*
Physical Activity (Units of accelaration, ENMO), mean (SD)	Objective	27.28 (9.93)	*r* = −*0.06, p* = *0.34*
Sleep efficiency score (Sleep Duration divided by Time in Bed, ranging from 0 to 1), mean (SD)	Objective	0.88 (0.09)	*r* = −*0.02, p* = *0.7*

Bold statistics indicate statistical significance results at alpha level 0.05.

To further illustrate the difference in smartphone application usage, we present the percentage of time spent on smartphone applications and phone calls in Supplementary Fig. 7. Most time was spent on instant messaging services and social media, followed by phone calls, with the least time dedicated to dating apps, and video chatting applications.

### Daily level mobile sensing, physical activity, sleep efficiency, social activity and loneliness

Firstly, we conducted a multilevel regression model with day level loneliness as the outcome (see Table 2). To ensure comparability, all variables were z-standardized, such that coefficients represent the change in daily loneliness per one standard deviation change in the predictor. To support interpretation, we also report results and their interpretation based on raw unstandardized values in Supplementary Table 4.

**Table 2:** Day Level Multilevel regression model

Predictors	Estimate	Std.error	Statistic	df	*p*- value	95% Bootstrapped CI	
Intercept	27.465	2.010	13.663	1842.306	<0.001	Lower	Upper
**Between-subject Effect**							
Sleep Efficiency	−0.752	1.152	−0.652	1768.463	0.514	−3.00	1.49
Physical Activity (ENMO)	−0.366	1.171	−0.313	1720.444	0.755	−2.63	1.93
**Social Interaction Duration**	−**5.546**	**1.144**	−**4.848**	**1857.511**	**<0.001**	−**7.76**	−**3.31**
Phone Call Duration	1.662	1.428	1.164	729.308	0.245	−1.06	4.49
SMS Length	1.397	1.418	0.986	642.803	0.325	−1.30	4.19
**Instant Messenger Duration**	**2.825**	**1.196**	**2.362**	**1856.254**	**0.018**	**0.51**	**5.17**
Video Chatting Duration	1.400	1.097	1.276	1906.341	0.202	−0.66	3.59
Social Media Duration	0.105	1.162	0.091	1897.894	0.928	−2.13	2.39
Dating App Duration	−0.175	1.095	−0.160	1910.820	0.873	−2.22	2.04
**Within-subject Effect**							
Sleep Efficiency	−0.275	0.409	−0.672	595.168	0.502	−1.04	0.49
Physical Activity (ENMO)	−0.733	0.403	−1.820	687.909	0.069	−1.46	0.04
**Social Interaction Duration**	−**2.923**	**0.393**	−**7.439**	**1354.738**	**<0.001**	−**3.65**	−**2.19**
Phone Call Duration	0.355	0.455	0.780	416.923	0.436	−0.51	1.20
SMS Length	0.219	0.435	0.504	504.009	0.614	−0.59	1.03
**Instant Messenger Duration**	**1.171**	**0.382**	**3.065**	**1688.024**	**0.002**	**0.47**	**1.88**
Video Chatting Duration	−0.031	0.364	−0.085	1917.920	0.932	−0.68	0.65
Social Media Duration	0.129	0.374	0.346	1818.137	0.729	−0.55	0.82
Dating App Duration	0.611	0.366	1.671	1869.425	0.095	−0.06	1.29
**Control Variables**							
Age	−0.791	1.132	−0.699	1692.333	0.485	−2.99	1.42
Gender [Male]	3.164	2.518	1.256	1866.891	0.209	−1.74	8.07
Gender [Diverse]	−2.378	10.506	−0.226	1872.130	0.821	−21.72	18.90
Weekday [Tuesday]	0.442	1.421	0.311	1517.422	0.756	−2.19	3.06
Weekday [Wednesday]	−0.478	1.429	−0.334	1466.174	0.738	−3.11	2.15
Weekday [Thursday]	−0.863	1.439	−0.599	1399.189	0.549	−3.50	1.78
Weekday [Friday]	−0.761	1.416	−0.537	1607.611	0.591	−3.39	1.84
Weekday [Saturday]	−2.260	1.419	−1.593	1638.887	0.111	−4.87	0.36
Weekday [Sunday]	−1.957	1.452	−1.348	1375.762	0.178	−4.63	0.73
**Lockdownstage [Lockdown]**	**5.470**	**2.179**	**2.511**	**1769.342**	**0.012**	**1.27**	**9.70**

Reference level: Gender = Female, Weekday = Monday, Lockdown= No-lockdown.

The outcome variable in this analysis is daily loneliness. We report the results based on multiple imputation (m = 100), including the estimated coefficients, standard errors, t-values, *p*-values, and 95% bootstrapped confidence intervals for each predictor. Predictors were considered statistically significant if their 95% bootstrapped confidence intervals (based on 1000 resamples) did not include zero. Statistically significant variables are highlighted in bold.

At the between-person level, greater average time spent in self-reported social interactions was associated with lower daily loneliness, B = –5.55, SE = 1.14, t(1857.51) = –4.85, *p* < 0.001, 95% Bootstrapped CI [–7.76, –3.31]. In contrast, higher average use of instant messaging was associated with increased daily loneliness, B = 2.83, SE = 1.20, t(1856.25) = 2.36, *p* = 0.018, 95% Bootstrapped CI [0.51, 5.17].

Within-person effects showed that on days when individuals engaged in more self-reported social interaction than usual, they reported significantly lower daily loneliness, B = –2.92, SE = 0.39, t(1354.74) = –7.44, *p* < .001, 95% Bootstrapped CI [–3.65, –2.19]. Conversely, greater daily use of instant messaging was associated with higher daily loneliness, B = 1.17, SE = 0.38, t(1688.02) = 3.07, *p* = 0.002, 95% Bootstrapped CI [0.47, 1.88]. Among control variables, being in a lockdown stage was associated with increased daily loneliness, B = 5.47, SE = 2.18, t(1769.34) = 2.51, *p* = 0.012, 95% Bootstrapped CI [1.27, 9.70], whereas age, gender, and weekday had no statistically significant effects (*ps* > .05, all 95% CIs included zero).

The following results differed statistically significantly between the imputed model and the listwise deletion model (see Supplementary Table 2). At the between-subject level, the bootstrapped model offered more robust evidence for a positive association between instant messaging and loneliness, whereas the listwise deletion model did not show a significant effect (B = 2.75, *p* = 0.079). This discrepancy likely reflects the increased statistical power and stability afforded by imputation. At the within-subject level, both models pointed in the same direction, but the bootstrapped model again provided stronger support for the link between instant messaging use and increased loneliness. While the listwise deletion model did not show a significant effect (B = 1.08, *p* = 0.080), this association reached statistical significance in the bootstrapped model.

### Momentary level mobile sensing, physical activity, sleep efficiency, social activity and loneliness

We conducted a multilevel regression model with momentary loneliness as the outcome (see Table 3). Again, all variables were z-standardized, such that coefficients represent the change in momentary loneliness per one standard deviation change in the predictor. To facilitate interpretation, we also report results and their interpretation based on raw values in Supplementary Table 4.

**Table 3:** Momentary Level Multilevel regression model

Predictors	Estimate	Std.error	Statistic	df	*p*- value	95% Bootstrapped CI	
Intercept	22.602	1.740	12.993	12,566.539	0.000	Lower	Upper
**Between-subject Effect**							
Sleep Efficiency	−0.573	1.185	−0.484	9371.037	0.629	−2.92	1.71
Physical Activity (ENMO)	−1.299	1.149	−1.130	10,641.163	0.258	−3.50	1.00
**Social Interaction Duration**	−**5.096**	**1.181**	−**4.316**	**12,294.356**	**<0.001**	−**7.40**	−**2.74**
Phone Call Duration	2.253	1.412	1.595	1237.304	0.111	−0.51	5.01
SMS Length	1.165	1.149	1.014	2717.642	0.310	−0.89	3.63
**Instant Messenger Duration**	**2.953**	**1.240**	**2.382**	**12,085.291**	**0.017**	**0.60**	**5.44**
Video Chatting Duration	1.267	1.143	1.108	12,660.220	0.268	−0.77	3.73
Social Media Duration	0.889	1.229	0.723	12,447.355	0.470	−1.46	3.35
**Within-subject Effect**							
**Sleep Efficiency**	−0.322	0.178	−1.811	758.673	0.070	−0.66	0.03
Physical Activity (ENMO)	−0.054	0.176	−0.307	1479.681	0.759	−0.39	0.29
**Social Interaction Duration**	−**2.564**	**0.155**	−**16.505**	**12,583.030**	**<0.001**	−**2.86**	−**2.26**
Phone Call Duration	0.014	0.191	0.073	751.333	0.942	−0.35	0.39
SMS Length	0.050	0.186	0.268	866.182	0.788	−0.30	0.42
**Instant Messenger Duration**	**0.736**	**0.153**	**4.792**	**12,722.019**	**<0.001**	**0.44**	**1.03**
Video Chatting Duration	0.292	0.153	1.904	12,727.584	0.057	0.01	0.60
**Social Media Duration**	**0.534**	**0.154**	**3.478**	**12,726.655**	**0.001**	**0.24**	**0.83**
**Control Variables**							
Age	−1.207	1.164	−1.037	12,306.814	0.300	−3.45	1.10
Gender [Male]	4.498	2.611	1.723	12,354.265	0.085	−0.56	9.67
Gender [Diverse]	4.781	10.963	0.436	12,497.165	0.663	−13.54	29.19
Weekday [Tuesday]	-0.558	0.573	−0.974	12,717.394	0.330	−1.67	0.55
**Weekday [Wednesday]**	−**1.773**	**0.572**	−**3.101**	**12,724.614**	**0.002**	−**2.87**	−**0.67**
Weekday [Thursday]	−0.750	0.577	−1.300	12,719.738	0.194	−1.86	0.37
Weekday [Friday]	−0.950	0.574	−1.655	12,726.150	0.098	−2.06	0.16
**Weekday [Saturday]**	−**3.108**	**0.580**	−**5.357**	**12,716.330**	**<0.001**	−**4.23**	−**1.98**
**Weekday [Sunday]**	−**2.252**	**0.579**	−**3.888**	**12,722.040**	**<0.001**	−**3.38**	−**1.13**
Lockdownstage [Lockdown]	−0.738	1.767	−0.418	12,661.809	0.676	−4.17	2.74

The outcome variable in this analysis is momentary loneliness. We report the results based on multiple imputation (m = 100), including estimated coefficients, standard errors, t-values, *p*-values, and 95% bootstrapped confidence intervals for each predictor. The 95% bootstrapped confidence intervals (based on 1000 resamples) are provided, with statistically significant variables highlighted in bold.

Reference level: Gender = Female, Weekday = Monday, Lockdown= No-lockdown.

At the between-person level, individuals who spent more average time in social interactions reported significantly lower momentary loneliness (*B* = –5.10, *SE* = 1.18, *t*(12,294.36) = –4.32, *p* < 0.001, 95% Bootstrapped CI [–7.40, –2.74]). Conversely, greater average use of instant messaging was associated with higher momentary loneliness (*B* = 2.95, *SE* = 1.24, *t*(12,085.29) = 2.38, *p* = 0.017, 95% Bootstrapped CI [0.60, 5.44]). Other between-person variables, such as sleep efficiency, physical activity, and other communication modes (e.g., phone calls, SMS, video chatting, and social media use), were not significantly related to loneliness (*ps* > 0.05; all 95% CIs included zero).

At the within-person level, participants reported significantly lower momentary loneliness at times when they engaged in more social interaction than they typically do, relative to their own average (*B* = –2.56, *SE* = 0.16, *t*(12,583.03) = –16.51, *p* < 0.001, 95% CI [–2.86, –2.26]). In contrast, greater use of instant messaging (*B* = 0.74, *SE* = 0.15, *t*(12,722.02) = 4.79, *p* < 0.001, 95% CI [0.44, 1.03]) and increased social media use (*B* = 0.53, *SE* = 0.15, *t*(12,726.66) = 3.48, *p* = 0.001, 95% CI [0.24, 0.83]), relative to the person’s own average, were both associated with significantly higher momentary loneliness. No significant within-person associations were found for sleep efficiency, physical activity, or other communication modes (*ps* > 0.05; all CIs included zero).

Among control variables, weekday effects emerged: momentary loneliness was significantly lower on Wednesday (B = –1.77, *p* = 0.002), Saturday (B = –3.11, *p* < 0.001), and Sunday (B = –2.25, *p* < 0.001) compared to Monday. No significant differences were observed for other weekdays. Gender, age, and lockdown stage were not significantly related to momentary loneliness.

The following results differed statistically significantly between the imputed model and the listwise deletion model (See Supplementary Table 2). At the between-person level, only the listwise model identified a significant effect of physical activity, with higher average activity levels associated with lower momentary loneliness (B = –3.86, *p* = 0.023). At the within-person level, the listwise deletion model found a significant negative association between sleep efficiency and momentary loneliness (B = –0.84, *p* < 0.001), suggesting that participants experienced less momentary loneliness on days when their sleep was more efficient than their own average.

### Temporal dynamic network estimation

**Fig. 3:**
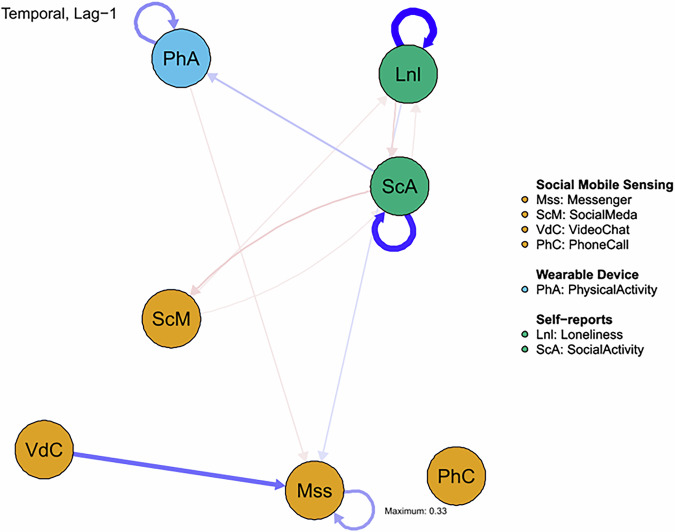
Temporal dynamic network to examine self-reported loneliness, social activity, and digital phenotyping. We analyzed the temporal relationships between self-reported loneliness, social activity, physical activity (as measured by actigraphy devices), and social mobile sensing, using a multilevel vector autoregressive model. These time lagged (lag 1) partial correlations are illustrated where variables are represented as nodes and edges (arrows connecting nodes) indicate statistically significant partial correlations (α < 0.05). Thicker and more saturated edges reflect stronger associations, with positive relationships represented in blue and negative relationships in red.

Figure 3 displays the temporal dynamic networks which include only statistically significant edges (i.e., time-lagged partial correlations with α < 0.05). The variables for dating app usage and SMS were excluded from the analysis due to an insufficient number of observations.

Momentary Loneliness was positively associated with subsequent messenger app usage (β = 0.04, *p* = 0.036), suggesting that higher momentary loneliness may drive increased text-based communication. Video chat use was also associated with increased subsequent use of messenger apps (β = 0.192, *p* = 0.010), suggesting that face-to-face digital interactions may prompt further text communication. Social media usage was associated with reduced momentary loneliness over time (β = −0.025, *p* = 0.002), but it was also linked to decreases in real-world social activity (β = −0.024, *p* = 0.044), indicating a complex relationship between digital and offline social engagement.

There was a significant negative association between physical activity and messenger usage (β = −0.030, *p* = 0.030), suggesting that individuals who engaged in more physical activity were less likely to use messenger apps at a subsequent timepoint. Strong autocorrelation effects were observed for physical activity (β = 0.147, *p* < 0.001), indicating that individuals tend to maintain consistent activity levels over time.

There was a significant negative time-lagged association between momentary loneliness and social activity (β = −0.046, *p* = 0.015), indicating that individuals who feel lonely tend to engage in fewer social activities afterwards. In turn, greater social activity was associated with reduced momentary loneliness (β = −0.026, *p* = 0.005), highlighting the reciprocal relationship between social engagement and emotional well-being. Greater social activity also positively predicted physical activity (β = 0.069, *p* < 0.001), suggesting that socially engaged individuals were more likely to be physically active. Increased social activity was associated with less subsequent social media use at subsequent timepoints (β = -0.053, *p* < 0.001). Strong autocorrelation effects were observed for both social activity (β = 0.270, *p* < 0.001) and momentary loneliness (β = 0.331, *p* < 0.001), suggesting that these behaviors tend to persist over time.

## Discussion

Digital phenotyping holds significant potential for identifying risk and protective factors of loneliness through smartphone-based mobile sensing and wearable actigraphy devices. In this study, we investigated how digital phenotyping relates to daily and momentary loneliness and estimated their temporal dynamics. More frequent real-world social interaction was consistently associated with lower loneliness, both at the daily and momentary levels, and across individuals as well as within individuals over time. In contrast, higher use of instant messaging and social media was associated with greater loneliness. Specifically, individuals who use instant messaging platforms more frequently than others, and those who surpass their own typical usage of instant messaging and social media, tend to experience higher levels of loneliness. Our exploratory temporal network analysis further suggests that physical activity was associated with real-life social interaction, which in turn is associated with reduced feelings of loneliness. Furthermore, loneliness is associated with more messenger apps usage, while social media usage is associated with decreased subsequent real-world social engagement. Overall, our results suggest that physical activity (measured by actigraphy), along with social media and instant messaging usage (measured through social mobile sensing) may serve as digital phenotyping markers of loneliness, indicating both protective and risk-related factors.

Our results indicate that individuals who use messenger apps more than others tend to report greater daily and momentary loneliness. Furthermore, when individuals use messenger apps more than they typically do, they also tend to experience heightened feeling of loneliness. This is in line with previous studies, reporting that increased usage time of smartphone communication apps was associated with lower social well-being and heightened feelings of loneliness^16,22^. Our exploratory temporal network analysis indicates that when people feel lonely, they may be more inclined to subsequently use communication apps on their smartphones to reach out to others. The underlying motives which drive smartphone usage may be key to explain this association. That is, whether individuals use social apps to strengthen and maintain existing relationships, such as arranging in-person meetings, or these apps serve as substitutes for real-world interactions^55^.

In addition, our results indicate that using social media platforms more than usual may intensify feelings of loneliness. Our exploratory network analysis further indicated that social media use is associated with more subsequent real-world social interactions, potentially contributing to this effect. This finding is in line with previous research, suggesting that individuals who use social media less frequently tend to experience higher levels of loneliness compared to those who are more actively engaged with social media^37,56^. At the same time, our exploratory network analysis indicates that social media use is also associated with reduced subsequent feeling of loneliness. This apparent contradiction may stem from differences between the short-term and long-term effects of social media. While social media engagement might temporarily reduce feelings of loneliness, it may, over time, contribute to greater loneliness by displacing real-world social interactions and reducing opportunities for deeper, face-to-face connection.

Additionally, our exploratory temporal dynamics analysis suggests that physical activity (PA) may indirectly reduce loneliness by being associated with more social interactions over time. This finding supports previous studies, indicating that PA reduces loneliness through social interactions during exercising^25^. However, we did not observe a direct effect of PA on daily or momentary levels of loneliness, which contrast with prior work. For example, a meta-analysis by Pels and Kleinert^24^ reported that PA was a significant direct predictor of loneliness. Similarly, several studies using passively sensed PA data from smartphones have found that higher activity levels are associated with lower loneliness^22,57,58^. However, in the listwise deletion model, we found that individuals who were more physically active on average reported lower levels of loneliness. This effect were not observed in the imputed data model, suggesting that such findings may depend on the subset of participants with complete information, particularly those with more consistent activity tracking^48^.

Contrary to our hypotheses, we found that other factors derived from social mobile sensing, such as phone call duration, the number of text messages, and the use of dating apps, or video calls, were not significantly associated with loneliness. This finding contrasts with a meta-review by Qirtas et al.^5^, which suggests that a range of social mobile sensing factors are associated with loneliness episodes. This discrepancy may be attributed to a lack of data on phone call and SMS length, and a lack of data variance in our sample, which was predominantly composed of users of social media and instant messaging apps.

Finally, we did not find sleep efficacy to be significantly associated with loneliness. This contrasts a previous a meta-analysis by Griffin et al.^30^, which reported a medium-sized correlation between loneliness and sleep disturbances. Furthermore, it has been suggested that poor sleep efficacy might lead to increased social withdrawal, which, in turn, could heighten feelings of loneliness^31^. However, the complete-case model indicated that participants experienced less loneliness on days when they slept better than usual. Again, this effect was not observed in the imputed data model, suggesting that such findings may depend on the subset of participants with complete information^48^.

Although digital phenotypes do not require active self-report from participants, they are not entirely free from subjectivity. Specifically, we did not explore the underlying motivations driving individuals to use social media, instant messaging, or engage in physical activity. For example, a person might use social media to maintain connections with friends, perhaps to arrange a future meeting, or may do so as a substitute for in-person interactions. Therefore, future studies should complement digital phenotyping with subjective self-reports to explore the underlying motivations behind smartphone usage. This would provide deeper insights into how different forms of digital phenotyping relate to loneliness.

We observed varying levels of missing data across measures, with particularly high rates for phone call and SMS length, as well as for actigraphy data. Additionally, some findings differed between the multiple imputation model and the listwise deletion model. In general, imputation models offer greater statistical power and help reduce bias due to missingness, resulting in more stable and representative estimates^48^. For this reason, we based our interpretation primarily on the imputed data. However, the listwise deletion model revealed significant effects of physical activity and sleep efficiency on loneliness, effects that were not present in the imputed model. This discrepancy may be because only a subset of participants, likely those more willing to wear sensors consistently, contributed to the complete data. As such, the listwise deletion results may reflect patterns specific to a non-representative group, limiting the generalizability of these findings.

Additionally, our study was conducted during the COVID-19 pandemic, which limits its generalizability to non-pandemic contexts. However, we did control our analysis for potential lockdown effects. Moreover, the smartphone application used in our study was compatible only with Android systems. This limitation may have affected our sample representativeness, as Android devices are more commonly used by men^59^. Another limitation is our measurement of loneliness. While daily loneliness was assessed using a 3-item short form of the UCLA scale, momentary loneliness was measured with a single-item scale. Two common criticisms of single-item scales are their questionable reliability and limited sensitivity due to a narrow scoring range. Despite these concerns, evidence supporting the effectiveness of single-item measures continues to grow^60^. Whether this is also true for loneliness is an important question that warrants further investigation in future studies.

Finally, future research could leverage social mobile sensing to determine optimal moments for delivering just-in-time adaptive interventions (JITAIs). These interventions rely on real-time data, such as ecological momentary assessments (EMA) of loneliness or passive mobile sensing, to assess an individual’s momentary state and trigger timely, and personalized support (e.g., prompting cognitive reappraisal or encouraging social engagement)^61^. Critical decision rules for triggering interventions could be based on behavioral patterns detected via mobile sensing, such as increased instant messaging or social media use, or decreased physical activity, which can signal heightened risk for loneliness.

In sum, we examined digital phenotyping indicators of loneliness using data from smartphones and wearable actigraphy devices, assessing associations at both daily and momentary levels and exploring their temporal dynamics. The primary digital phenotyping factor associated with daily loneliness was messenger app usage. Moreover, social media and messenger app use were linked to momentary loneliness. An exploratory temporal dynamic network analysis indicates that social interactions increase subsequent physical activity and reduce feelings of loneliness. Moreover, our findings suggest that experiences of loneliness may prompt individuals to subsequently engage more with communication apps on their smartphones. More frequent use of social media apps was associated with reduced real-world social interaction, which may, in turn, increases feelings of loneliness. In summary, digital phenotyping can be a valuable approach for estimating loneliness in a passive, continuous, and objective manner. Key risk factors for loneliness derived from social mobile sensing include decreased physical activity, increased social media engagement, and more frequent use of instant messaging.

## Supplementary information

Supplementary Information

## Data Availability

The data and R code used for the statistical analyses can be found online: https://osf.io/rs2nd/.

## Code availability

The R code used for the statistical analyses can be found online: https://osf.io/rs2nd/.
