# Supplementary material for: Objective risk and protective factors for momentary and daily loneliness:using digital phenotyping and temporal analysis
Source: Npj Ment Health Res. 2025 Sep 7;4:42. doi: 10.1038/s44184-025-00148-4 (PMC12413993; doi:10.1038/s44184-025-00148-4)
Supplement: Supplementary file 1 — Supplementary Information [file 44184_2025_148_MOESM1_ESM.pdf]

Supplementary Information

Supplementary Figure 1. Missing Data. Panel A displays the percentage of missing data by variable in the day-level dataset, and Panel B shows the same for the momentary-level dataset. In total, 7.8% of the day-level data and 7% of the momentary-level data were missing, with 92.2% and 93% of the data fully observed, respectively.

A.

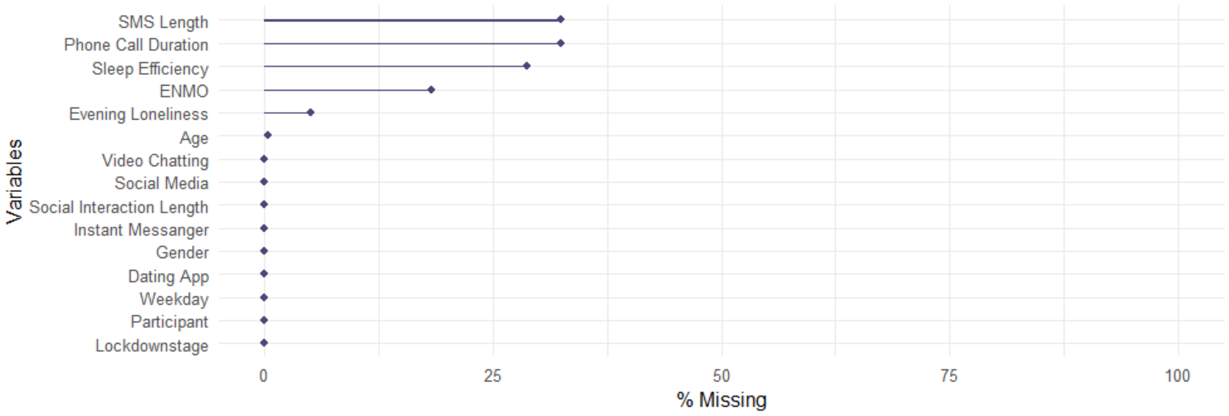

B.

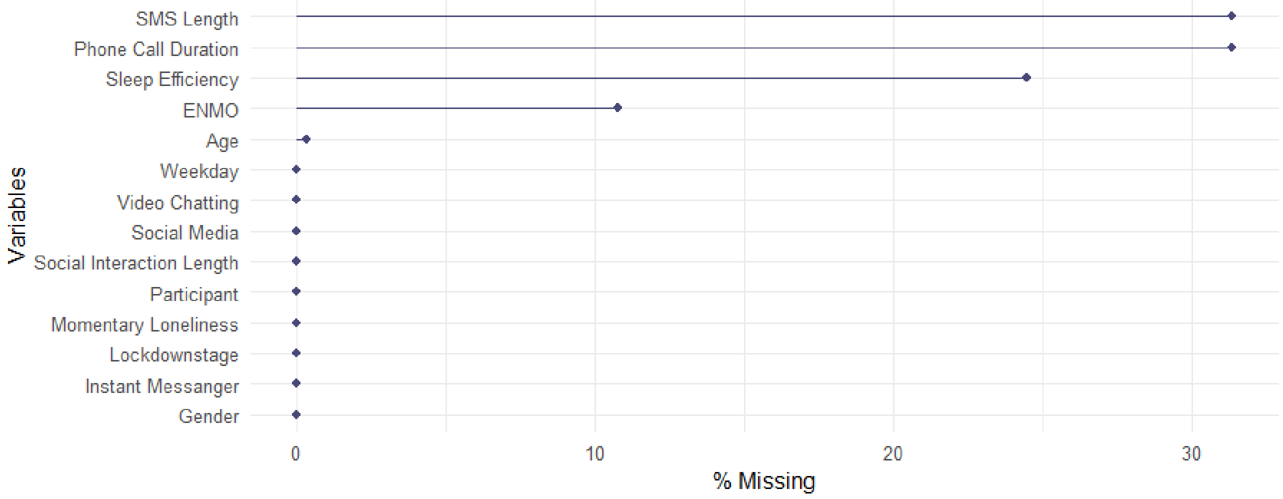

Supplementary Figure 2. Distribution of imputed data.

A. Day Level Imputation.

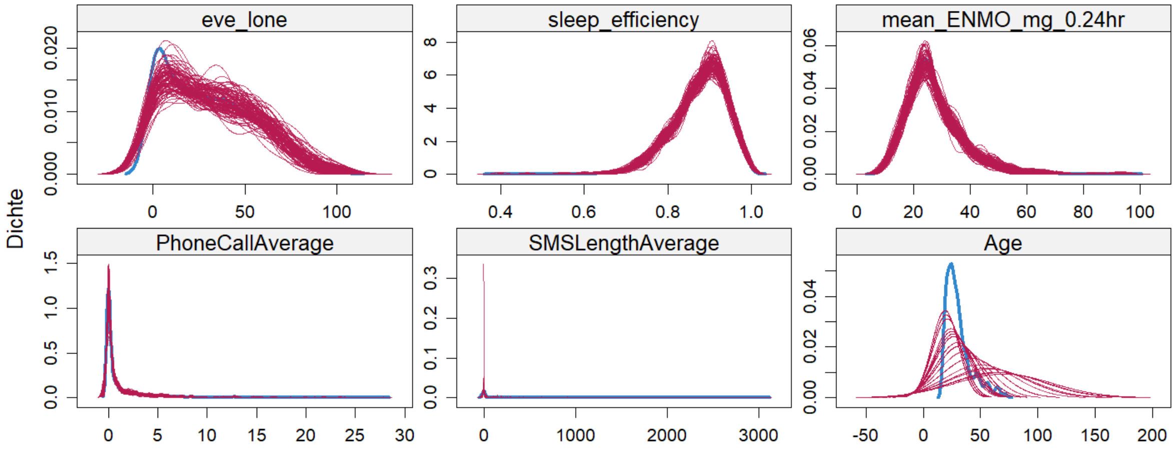

B. Momentary Level Imputation.

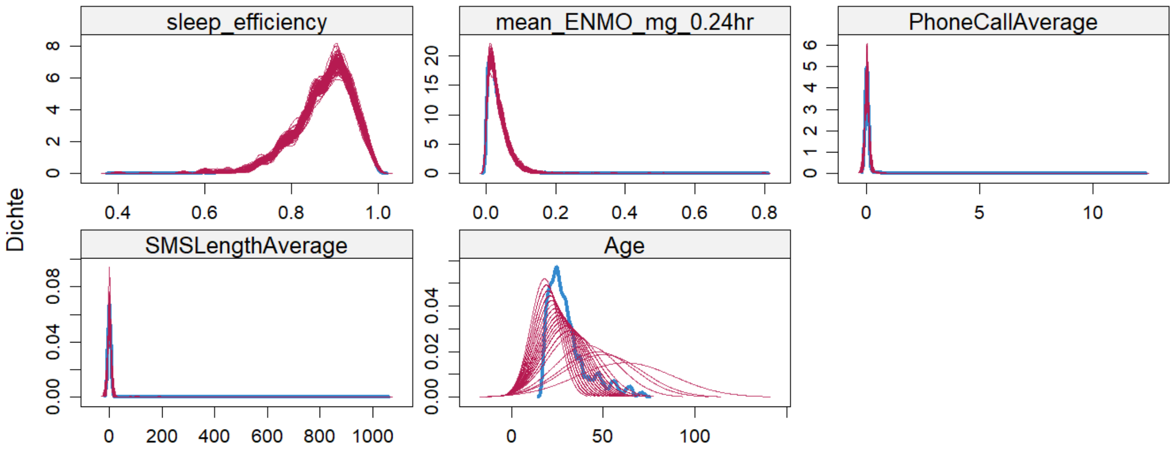

Supplementary Figure 3. Assumption check. Day Level Analysis

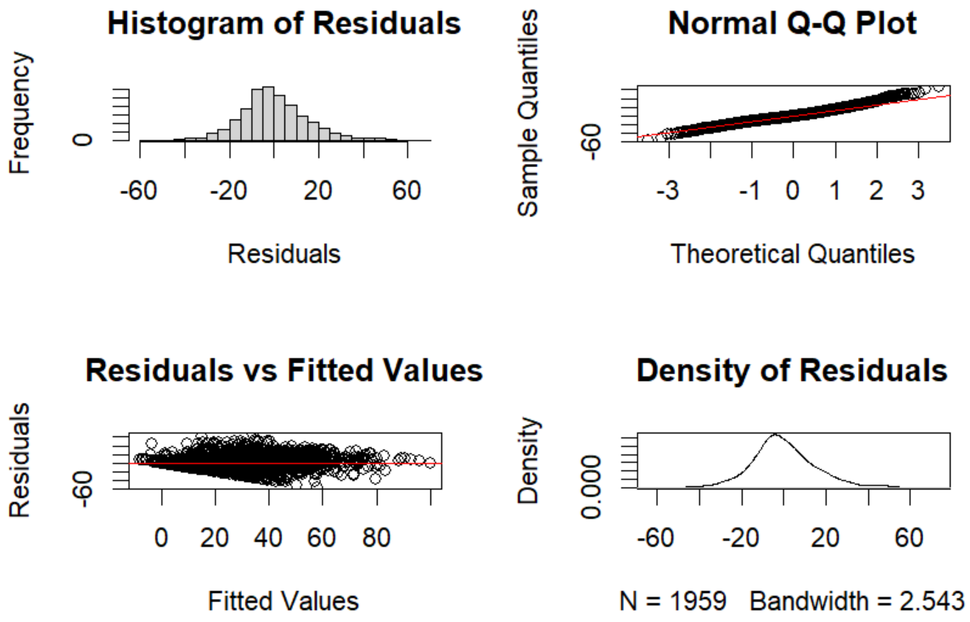

Momentary Level Analysis

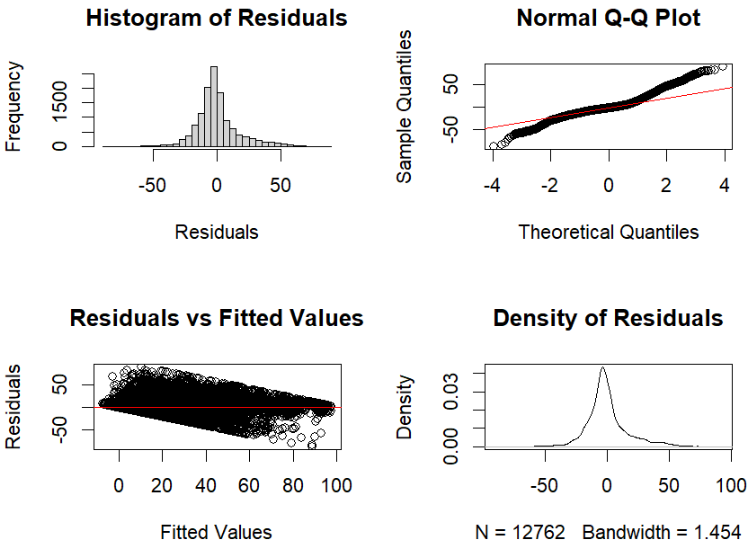

Supplementary Figure 4. Outlier exclusion

A. Outlier exclusion of ENMO Scores. A. Boxplot showing ENMO scores prior to the exclusion of 3 outliers. B. Boxplot showing ENMO scores after excluding 3 outliers (2> SD above the mean)

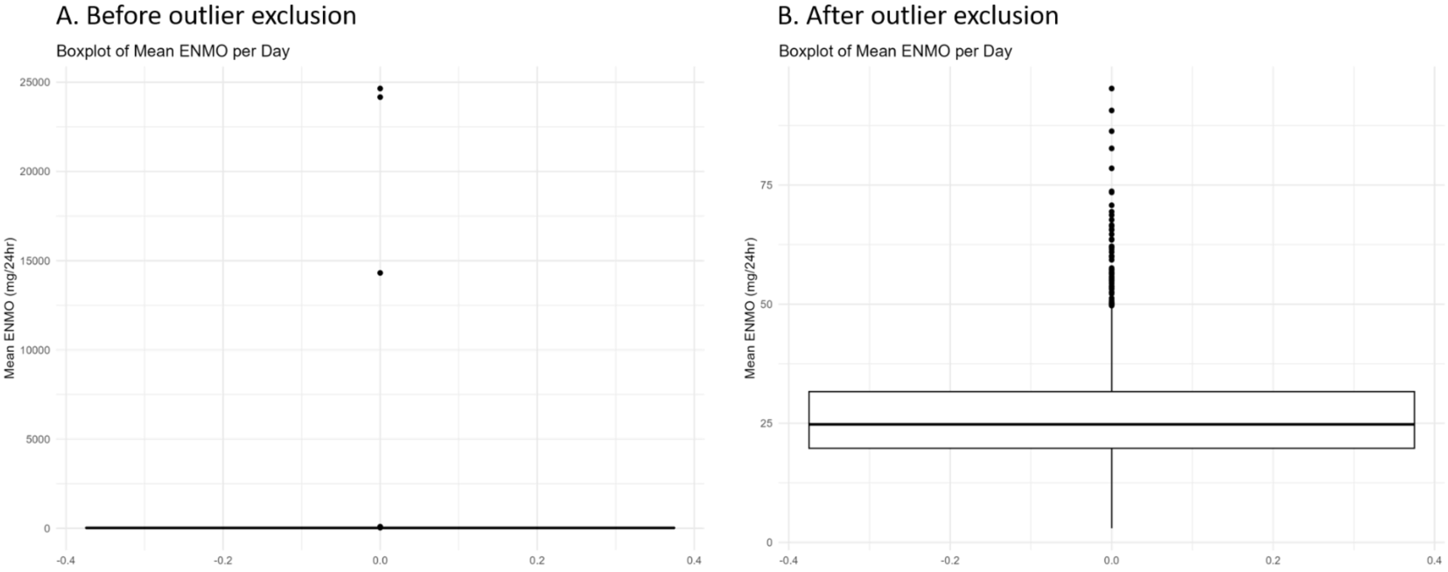

B. Outlier exclusion of Sleep Duration Scores. A. Boxplot showing Sleep Duration prior to the exclusion of 56 outliers. B. Boxplot showing Sleep Duration after excluding 56 outliers.

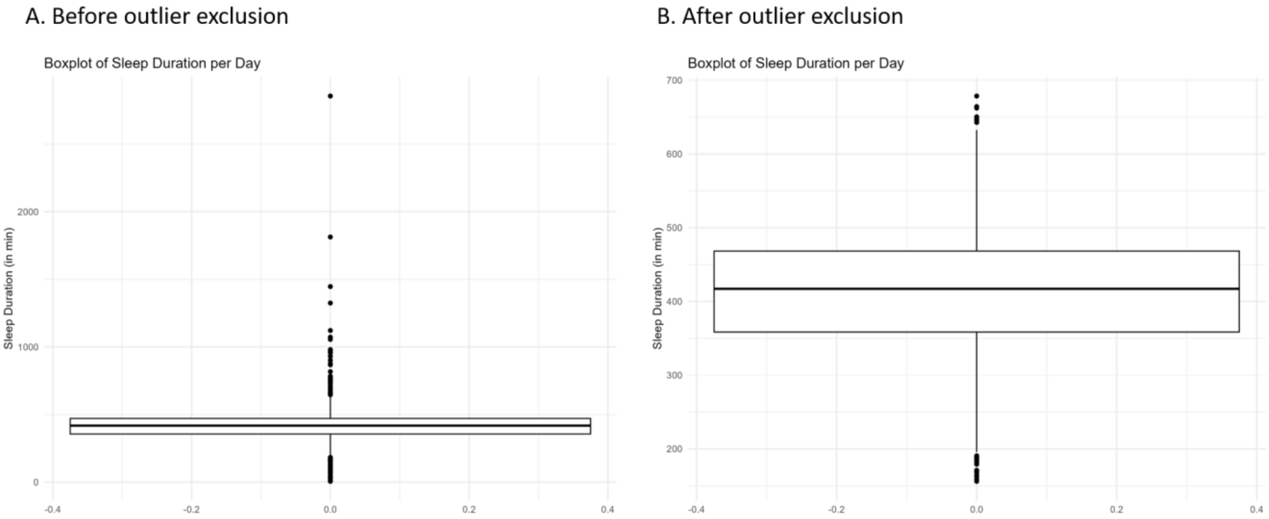

Supplementary Figure 5. Calculation of momentary levels of social mobile sensing and physical activity.

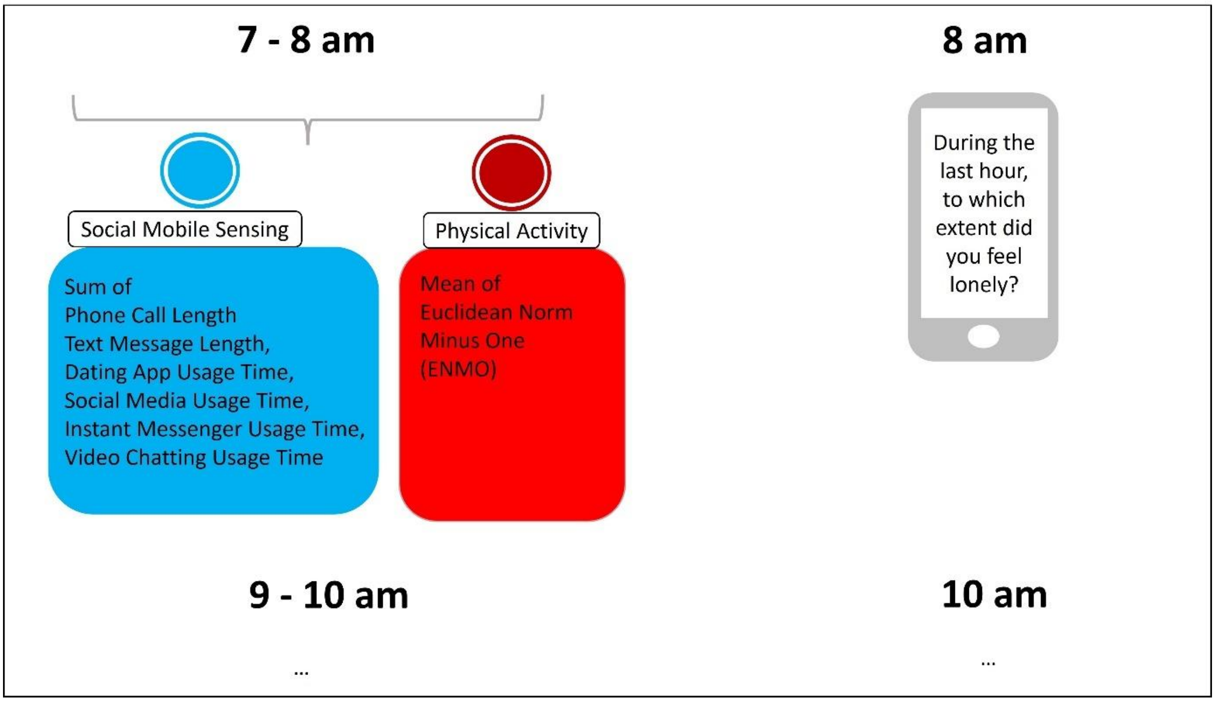

Supplementary Figure 6. Distribution of data points one hour before each momentary beep.

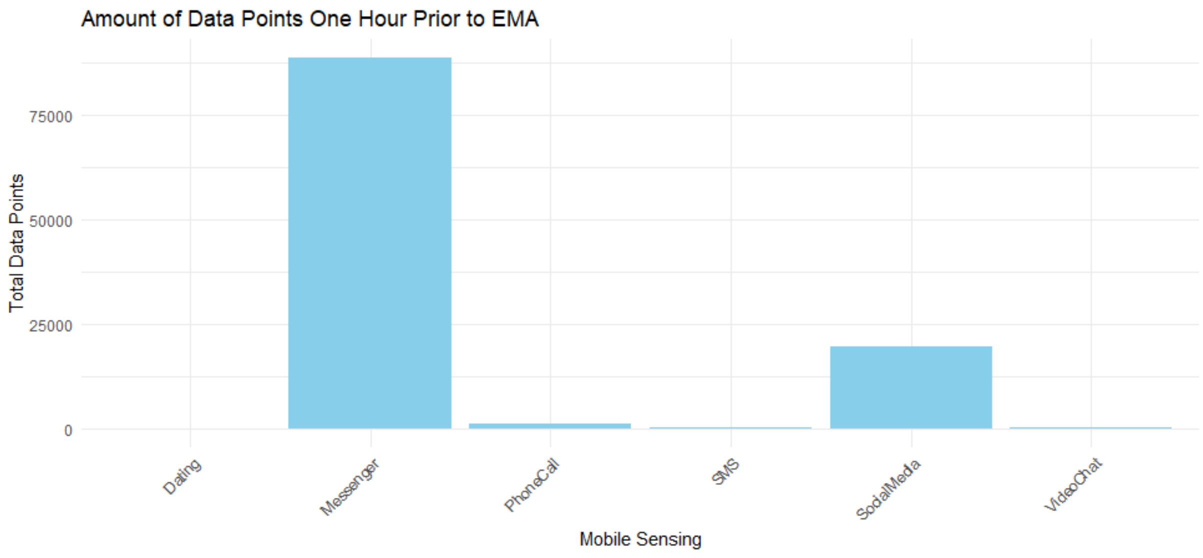

Supplementary Figure 7. Group comparisons between high and low anxiety group (before imputation). In both groups, most time was spent on instant messaging services and social media, followed by phone calls, with the least time dedicated to dating apps, and video chatting applications. To display differences among individuals experiencing loneliness, we performed a median split via the UCL-3 loneliness scores. This categorized participants into two groups: High Loneliness and Low Loneliness.

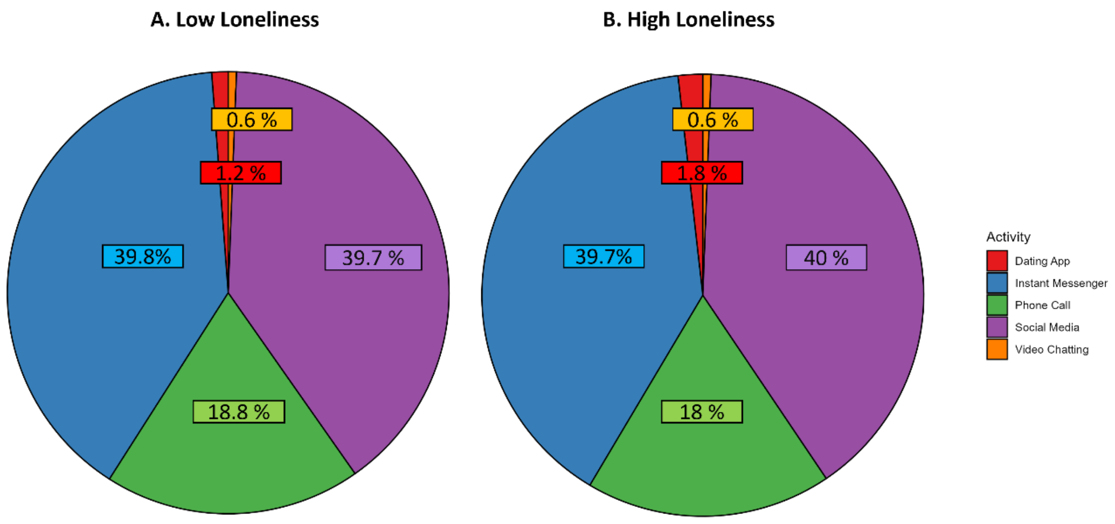

Supplementary Table 1. Model selection procedure (based on list-wise deletion model).

Day Level Analysis

Model:	AIC
**Random intercept model, fixed slopes**	**8067.5**
Random intercept model, random slope for Sleep Efficiency (Between Subjects)	8071.5

Momentary Level Analysis

Model:	AIC

**Random intercept model, fixed slopes**	**55210**
Random intercept model, random slope for Messenger App usage	55214

Supplementary Table 2. List-wise deletion results

A. Results of list-wise deletion of daily loneliness. Bold statistics indicate statistical significance results at alpha level 0.05.

	**Day-level loneliness**				
Predictors	Estimates	std. Error	Statistic	p	df
(Intercept)	27.28	2.72	10.02	**<0.001**	902.00
**Between-subjects effect**					
Sleep Efficiency	-1.09	1.52	-0.71	0.475	902.00
Physical Activity (ENMO)	-0.88	1.56	-0.57	0.571	902.00
**Social Interaction Duration**	-7.37	1.74	-4.24	**<0.001**	902.00
Phone Call Duration	0.30	1.59	0.19	0.851	902.00
SMS Length	1.83	1.48	1.23	0.219	902.00
Instant Messenger Duration	2.75	1.56	1.76	0.079	902.00
Video Chatting Duration	0.63	1.50	0.42	0.675	902.00
Social Media Duration	1.18	1.54	0.77	0.443	902.00
Dating App Duration	-0.21	1.38	-0.15	0.878	902.00
**Within-subjects effects**					
Sleep Efficiency	0.00	0.47	0.01	0.992	902.00
Physical Activity (ENMO)	-0.70	0.47	-1.48	0.139	902.00
**Social Interaction Duration**	**-3.48**	**0.52**	**-6.71**	**<0.001**	**902.00**
Phone Call Duration	0.07	0.56	0.13	0.893	902.00
SMS Length	0.20	0.47	0.44	0.660	902.00
Instant Messenger Duration	1.08	0.62	1.75	0.080	902.00

Video Chatting Duration	-0.34	0.60	-0.56	0.576	902.00
Social Media Duration	-0.43	0.48	-0.90	0.368	902.00
Dating App Duration	0.58	0.55	1.04	0.299	902.00
**Control variables**					
Age	-1.64	1.56	-1.05	0.295	902.00
Gender [Male]	2.08	3.37	0.62	0.537	902.00
Weekday [Tuesday]	-1.18	1.82	-0.65	0.516	902.00
Weekday [Wednesday]	-2.51	1.81	-1.38	0.167	902.00
**Weekday [Thursday]**	**-3.94**	**1.84**	**-2.14**	**0.033**	**902.00**
Weekday [Friday]	-3.01	1.90	-1.58	0.113	902.00
**Weekday [Saturday]**	**-6.21**	**1.93**	**-3.21**	**0.001**	**902.00**
Weekday [Sunday]	-2.73	1.87	-1.46	0.144	902.00
**Lockdownstage [Lockdown]**	**9.24**	**3.02**	**3.06**	**0.002**	**902.00**
Random Effects					
σ^2	210.30				
$\tau_{00\ Participant}$	317.52				
ICC	0.60				
$N_{Participant}$	173				
Observations	932				
Marginal R^2 / Conditional R^2	0.157 / 0.664				

B. Results of list-wise deletion of momentary loneliness. Bold statistics indicate statistical significance results at alpha level 0.05.

	Momentary loneliness				
Predictors	Estimates	std. Error	Statistic	p	df
Intercept	21.33	2.51	8.49	**<0.001**	6456.00
**Between-person**					
Sleep Efficiency	0.36	1.62	0.22	0.824	6456.00

**Physical Activity (ENMO)**	**-3.86**	**1.69**	**-2.28**	**0.023**	**6456.00**
**Social Interaction Duration**	**-4.77**	**1.83**	**-2.60**	**0.009**	**6456.00**
Phone Call Duration	0.33	1.89	0.17	0.863	6456.00
SMS Length	1.24	1.29	0.96	0.337	6456.00
Instant Messenger Duration	3.20	1.68	1.90	0.057	6456.00
Video Chatting Duration	1.55	1.52	1.02	0.309	6456.00
Social Media Duration	1.28	1.74	0.74	0.461	6456.00
**Within-person**					
**Sleep Efficiency**	-0.84	0.20	-4.20	**<0.001**	6456.00
Physical Activity (ENMO)	0.19	0.19	0.97	0.334	6456.00
**Social Interaction Duration**	-2.57	0.21	-12.47	**<0.001**	6456.00
Phone Call Duration	0.09	0.22	0.43	0.664	6456.00
SMS Length	0.01	0.19	0.06	0.952	6456.00
**Instant Messenger Duration**	1.12	0.22	5.14	**<0.001**	6456.00
Video Chatting Duration	0.30	0.20	1.49	0.136	6456.00
**Social Media Duration**	0.48	0.19	2.50	**0.013**	6456.00
**Control Variables**					
Age	-1.15	1.72	-0.67	0.504	6456.00
Gender [Male]	2.98	3.65	0.82	0.414	6456.00
Weekday [Tuesday]	-1.17	0.77	-1.53	0.127	6456.00
**Weekday [Wednesday]**	**-2.42**	**0.76**	**-3.17**	**0.002**	**6456.00**
**Weekday [Thursday]**	**-1.55**	**0.77**	**-2.00**	**0.046**	**6456.00**
Weekday [Friday]	-1.33	0.80	-1.66	0.097	6456.00
**Weekday [Saturday]**	-4.56	0.81	-5.65	**<0.001**	6456.00
**Weekday [Sunday]**	-2.27	0.78	-2.90	**0.004**	6456.00
Lockdownstage [Lockdown]	1.32	2.81	0.47	0.639	6456.00
Random Effects					
σ^2	261.11				

τ_{00} Participant	415.30
ICC	0.61
N Participant	171
Observations	6484
Marginal R^2 / Conditional R^2	0.102 / 0.653

Supplementary Table 3. Description of the Meta data. Meta data with examples and the information derived for the study.

Data type	Raw information	Example log entry	Information derived
App Usage	Time since start of assessment, Current App	4411, App, com.whatsapp/Converstation.	Name and type of applications, and length of use
Phone Call	Time since start of assessment, Type of call, hashed number, diration, time and date	442637,Call,type=Outgoing|number={"ONE_WAY_HASH*": "1c3d3074811843e1e133a3cba16d506ecb7e8593"} |duration=121|time=13:44:53|date=2020-10-01	Duration of phone calls
SMS Activity	Time since start of assessment, Type of call, hashed number, diration, time and date	32625,Call,type=Outgoing|number={"ONE_WAY_HASH": "1c3d3074811843e1e133a3cba16d506ecb7e8593"} |length=52|time=19:51:36|date=2020-09-01	Length of text messages

*this logs the phone activity in an anonymized way (Hashing of phone numbers)

Supplementary Table 4. Day Level Analysis. Unstandardized Results. Bold statistics indicate statistical significance results at alpha level 0.05.

term	estimate	std.error	statistic	df	p.value
(Intercept)	48.861	20.896	2.338	1,770.316	0.019
Between-subjects Effect					
Sleep Efficiency	-14.610	22.971	-0.636	1,786.227	0.525
Physical Activity (ENMO)	-0.044	0.143	-0.309	1,743.320	0.757
**Social Interaction Duration**	-5.418	1.147	-4.725	1,863.601	0.000
Phone Call Duration	0.872	0.752	1.159	868.155	0.247
SMS Length	0.009	0.009	0.967	747.271	0.334
**Instant Messenger Duration**	0.826	0.359	2.303	1,866.499	0.021
Video Chatting Duration	5.917	4.736	1.249	1,908.650	0.212
Social Media Duration	0.029	0.324	0.089	1,900.979	0.929
Dating App Duration	-0.395	2.564	-0.154	1,912.422	0.878
Within-subjects effect					
Sleep Efficiency	-5.624	8.853	-0.635	512.455	0.526
Physical Activity (ENMO)	-0.103	0.061	-1.689	591.199	0.092
**Social Interaction Duration**	-2.999	0.404	-7.430	1,358.117	0.000
Phone Call Duration	0.151	0.198	0.763	406.329	0.446
SMS Length	0.001	0.003	0.492	494.503	0.623
**Instant Messenger Duration**	0.494	0.162	3.055	1,691.509	0.002
Video Chatting Duration	-0.064	0.782	-0.081	1,917.539	0.935
Social Media Duration	0.062	0.173	0.357	1,818.289	0.721
Dating App Duration	1.335	0.804	1.660	1,869.503	0.097
**Control variables**					
Age	-0.070	0.103	-0.676	1,709.809	0.499

term	estimate	std.error	statistic	df	p.value
Gender [Male]	3.170	2.582	1.228	1,873.787	0.220
Gender [Diverse]	-2.329	10.767	-0.216	1,877.928	0.829
Weekday [Tuesday]	0.448	1.427	0.314	1,522.539	0.754
Weekday [Wednesday]	-0.481	1.435	-0.335	1,470.817	0.738
Weekday [Thursday]	-0.862	1.445	-0.597	1,406.997	0.551
Weekday [Friday]	-0.760	1.422	-0.535	1,611.776	0.593
Weekday [Saturday]	-2.274	1.424	-1.596	1,644.236	0.111
Weekday [Sunday]	-1.949	1.457	-1.337	1,382.117	0.181
**Lockdownstage [Lockdown]**	5.432	2.231	2.435	1,770.355	0.015

Between-subjects level. At the between-person level, greater average time spent in self-reported social interactions was associated with lower daily loneliness, $B = –5.42$, $SE = 1.15$, $t(1863.60) = –4.73$, $p < .001$. Specifically, a 10-minute increase in average daily social interaction was associated with an approximately 5-point reduction in loneliness on a visual analog scale ranging from 0 (not at all lonely) to 100 (extremely lonely). Additionally, higher average use of instant messaging was also significantly associated with increased loneliness, $B = 0.83$, $SE = 0.36$, $t(1866.50) = 2.30$, $p = .021$. Specifically, a 10-minute increase in average daily instant messaging was associated with an approximately 1-point increase in loneliness on a visual analog scale ranging from 0 (not at all lonely) to 100 (extremely lonely). No other between-person effects (e.g., sleep efficiency, physical activity, or other digital social interaction types) were statistically significant (*ps* > .05).

Within-subjects level. On days when individuals engaged in more self-reported social interaction than usual, they reported significantly lower loneliness, $B = –3.00$, $SE = 0.40$, $t(1358.12) = –7.43$, $p < .001$. That is, a 10-minute increase in self-reported social interaction, relative to an individual's own daily average, was associated with an approximate 3-point decrease in loneliness on a visual analog scale ranging from 0 (not at all lonely) to 100 (extremely lonely). Similarly, greater daily use of instant messaging was linked to higher loneliness, $B = 0.49$, $SE = 0.16$, $t(1691.51) = 3.06$, $p = .002$. That is, a 10-minute increase in use of instant messaging, relative to an individual's own daily average, was associated with an approximate 0.5-point decrease in loneliness on a visual analog scale ranging from 0 (not at all lonely) to 100 (extremely lonely). No other within-person effects reached significance (*ps* > .05), though the association between physical activity and loneliness approached significance ($p = .092$).

Control variables. Among control variables, being in a lockdown stage was significantly associated with increased loneliness, compared to not being in a lockdown stage ($B = 5.43$, $SE = 2.23$, $t(1770.36) = 2.44$, $p = .015$). Age, gender, and weekday showed no significant effects (*ps* > .05).

Momentary Level Analysis. Unstandardized Results. Bold statistics indicate statistical significance results at alpha level 0.05.

term	estimate	std.error	statistic	df	p.value
(Intercept)	43.535	21.639	2.012	9,929.118	0.044
**Between-subjects effects**					
Sleep Efficiency	-11.546	24.419	-0.473	9,704.775	0.636
Physical Activity (ENMO)	-106.262	95.883	-1.108	10,879.246	0.268
**Social Interaction Duration**	-5.079	1.203	-4.220	12,386.092	0.000
Phone Call Duration	21.428	13.618	1.573	1,441.135	0.116
SMS Length	0.102	0.101	1.007	3,453.705	0.314
**Instant Messenger Duration**	14.862	6.385	2.328	12,181.723	0.020
Video Chatting Duration	73.299	67.523	1.086	12,669.459	0.278
Social Media Duration	4.426	6.257	0.707	12,477.211	0.479
**Within-subjects effects**					
Sleep Efficiency	-6.486	3.908	-1.659	634.659	0.098
Physical Activity (ENMO)	-1.532	5.048	-0.303	1,464.161	0.762
**Social Interaction Duration**	-1.266	0.077	-16.497	12,581.076	0.000
Phone Call Duration	0.028	0.386	0.073	760.102	0.942
SMS Length	0.001	0.005	0.267	860.329	0.789
**Instant Messenger Duration**	1.848	0.386	4.786	12,721.964	0.000
Video Chatting Duration	3.266	1.715	1.904	12,727.358	0.057
**Social Media Duration**	1.379	0.397	3.474	12,726.539	0.001
**Control variables**					
Age	-0.108	0.107	-1.015	12,356.297	0.310
Gender [Male]	4.501	2.672	1.685	12,400.910	0.092
Gender [Diverse]	4.780	11.213	0.426	12,528.505	0.670
Weekday [Tuesday]	-0.555	0.574	-0.967	12,716.329	0.334

term	estimate	std.error	statistic	df	p.value
**Weekday [Wednesday]**	-1.771	0.572	-3.096	12,724.175	0.002
Weekday [Thursday]	-0.748	0.577	-1.296	12,718.786	0.195
Weekday [Friday]	-0.951	0.574	-1.656	12,725.919	0.098
**Weekday [Saturday]**	-3.114	0.581	-5.362	12,715.019	0.000
**Weekday [Sunday]**	-2.251	0.579	-3.885	12,721.944	0.000
**Lockdownstage [Lockdown]**	-0.777	1.790	-0.434	12,666.333	0.664

Between-subjects level. At the between-person level, individuals who spent more time on average in self-reported social interactions reported significantly lower momentary loneliness, $B = –5.08$, $SE = 1.20$, $t(12,386.09) = –4.22$, $p < .001$. Specifically, a 10-minute increase in average social interaction was associated with an approximate 5-point decrease in loneliness on a visual analog scale ranging from 0 (*not at all lonely*) to 100 (*extremely lonely*). Moreover, individuals who spent more time on average on instant messenger services reported significantly higher momentary loneliness, $B = 14.87$, $SE = 6.39$, $t(12,181.723) = 2.33$, $p < .001$. This between-subjects effect indicates that a 10-minute increase in a person's typical (average) instant messaging use was associated with an approximately 15-point increase in momentary loneliness on a 0–100 visual analog scale. No other between-person predictors (e.g., sleep efficiency, physical activity, or other digital communication modalities) were statistically significant (*ps* > .05).

Within-subjects level. At the momentary within-person level, individuals reported significantly lower loneliness at times when they engaged in more social interaction than usual, $B = –1.27$, $SE = 0.08$, $t(12,581.08) = –16.50$, $p < .001$. That is, a 10-minute increase in social interaction, relative to a person's own average, was associated with an approximate 1.3-point reduction in momentary loneliness. Conversely, increased use of instant messaging at a given moment was associated with significantly greater loneliness, $B = 1.85$, $SE = 0.39$, $t(12,721.96) = 4.79$, $p < .001$, equivalent to an increase of about 1.8 points in loneliness per 10 minutes of messaging. Momentary increases in social media use were also associated with higher loneliness, $B = 1.38$, $SE = 0.40$, $t(12,726.54) = 3.47$, $p = .001$ equivalent to an increase of about 1.4 points in loneliness per 10 minutes of social media usage. No other within-person predictors were statistically significant (*ps* > .05), though the effect of video chatting approached significance ($p = .057$).

Control variables. None of the demographic or contextual control variables, including age, gender, and lockdown status, were significantly associated with momentary loneliness (*ps* > .05), with the exception of weekday effects. Loneliness was significantly lower on **Wednesdays** ($B = –1.77$, $p = .002$), **Saturdays** ($B = –3.11$, $p < .001$), and **Sundays** ($B = –2.25$, $p < .001$), compared to Mondays.
